# Investigation of Genetic Variants, Birthweight and Hypothalamic-Pituitary-Adrenal Axis Function Suggests a Genetic Variant in the SERPINA6 Gene Is Associated with Corticosteroid Binding Globulin in the Western Australia Pregnancy Cohort (Raine) Study

**DOI:** 10.1371/journal.pone.0092957

**Published:** 2014-04-01

**Authors:** Laura N. Anderson, Laurent Briollais, Helen C. Atkinson, Julie A. Marsh, Jingxiong Xu, Kristin L. Connor, Stephen G. Matthews, Craig E. Pennell, Stephen J. Lye

**Affiliations:** 1 Lunenfeld-Tanenbaum Research Institute, Mount Sinai Hospital, Toronto, Ontario, Canada; 2 School of Women's and Infants' Health, The University of Western Australia, Crawley, Western Australia, Australia; 3 Department of Physiology, University of Toronto, Toronto, Ontario, Canada; 4 Fraser Mustard Institute of Human Development and Department of Obstetrics and Gynaecology, University of Toronto, Toronto, Ontario, Canada; Harvard Medical School, United States of America

## Abstract

**Background:**

The hypothalamic-pituitary-adrenal (HPA) axis regulates stress responses and HPA dysfunction has been associated with several chronic diseases. Low birthweight may be associated with HPA dysfunction in later life, yet human studies are inconclusive. The primary study aim was to identify genetic variants associated with HPA axis function. A secondary aim was to evaluate if these variants modify the association between birthweight and HPA axis function in adolescents.

**Methods:**

Morning fasted blood samples were collected from children of the Western Australia Pregnancy Cohort (Raine) at age 17 (n = 1077). Basal HPA axis function was assessed by total cortisol, corticosteroid binding globulin (CBG), and adrenocorticotropic hormone (ACTH). The associations between 124 tag single nucleotide polymorphisms (SNPs) within 16 HPA pathway candidate genes and each hormone were evaluated using multivariate linear regression and penalized linear regression analysis using the HyperLasso method.

**Results:**

The penalized regression analysis revealed one candidate gene SNP, rs11621961 in the CBG encoding gene (*SERPINA6*), significantly associated with total cortisol and CBG. No other candidate gene SNPs were significant after applying the penalty or adjusting for multiple comparisons; however, several SNPs approached significance. For example, rs907621 (p = 0.002) and rs3846326 (p = 0.003) in the mineralocorticoid receptor gene (*NR3C2*) were associated with ACTH and *SERPINA6* SNPs rs941601 (p = 0.004) and rs11622665 (p = 0.008), were associated with CBG. To further investigate our findings for *SERPINA6*, rare and common SNPs in the gene were imputed from the 1,000 genomes data and 8 SNPs across the gene were significantly associated with CBG levels after adjustment for multiple comparisons. Birthweight was not associated with any HPA outcome, and none of the gene-birthweight interactions were significant after adjustment for multiple comparisons.

**Conclusions:**

Our study suggests that genetic variation in the SERPINA6 gene may be associated with altered CBG levels during adolescence. Replication of these findings is required.

## Introduction

Early life exposures (both intrauterine and early post-natal) have a profound impact on predisposition to adult chronic diseases, including type II diabetes, hypertension, heart disease, stroke and neurologic disorders [1], [2], [3]. This area of research, often referred to as Developmental Origins of Health and Disease (DOHaD), was first supported by evidence that low birthweight is associated with coronary heart disease [4]. The hypothalamic-pituitary-adrenal (HPA) axis regulates stress response and HPA dysfunction has been associated with obesity, hypertension, depression, cardiovascular disease and other chronic diseases [5], [6], [7]. Evidence from laboratory studies suggests that premature activation of the fetal HPA axis is a central component linking adverse pre and/or post-natal environmental exposures to later poor health outcomes [8], [9], [10] and may underlie DOHaD; however, there is limited research from human studies.

Birth size reflects the fetal environment and low birthweight, a consequence of an adverse uterine environment and suboptimal growth, has been shown to alter the HPA axis [11]. Birthweight has been associated with HPA activity, primarily cortisol levels, in humans [12], [13], [14], [15], [16], [17]; however, this has not been found in all studies of children [18], [19] or adults [20]. Young children exposed to adverse environments post conception have also been found to have altered stress responses [21].

There are several genes involved in regulating the HPA axis, yet relatively few human studies have comprehensively evaluated polymorphisms in these genes and their associations with HPA activity, including cortisol, corticosteroid binding globulin (CBG), and adrenocorticotropic hormone (ACTH) concentrations [22]. Cortisol concentrations have been evaluated most frequently and polymorphisms in genes including the FK506 binding protein (*FKBP5*) [23], glucocorticoid receptor (*NR3C1*) [24], mineralcorticoid receptor (*NR3C2*) and pro-opiomelanocortin (*POMC*) [24] have been associated with cortisol levels in adults. Haplotypes of the glucocorticoid receptor have been found to modify the association between birth length and fasting plasma cortisol in older adults [25].

The objectives of this study were to evaluate whether tagging polymorphisms within 16 genes that contribute to HPA axis function were associated with 5 measures of basal HPA axis function (saliva cortisol, and plasma measures of total cortisol, ACTH, CBG and calculated free cortisol). The secondary objective was to evaluate if these genetic variants modify the association between birthweight and HPA axis function among adolescents in the Raine cohort study.

## Methods

### Western Australia Pregnancy Cohort (Raine) Study

Data were collected as part of the Raine cohort study. As described previously [26], [27], the Raine Study recruited 2,900 pregnancies from King Edward Memorial Hospital (KEMH) between 1989 and 1991 as part of a randomised controlled trial which evaluated repeated ultrasounds in pregnancy. Detailed data were collected throughout pregnancy [28] and at follow-ups carried out at ages 1, 2, 3, 6, 8, 10, 14 and 17 years [26], [28], [29].

There were 2,168 participants still enrolled in the cohort at 17 years of age and 1,754 agreed to participate in the 17 year follow-up. Of these individuals, 1,408 provided informed consent to take part in the basal cortisol study. Based on study criteria 331 individuals were excluded: 191 with at least one parent not of European descent, 11 currently or recently pregnant, 52 from twin or multiple births, 28 with a sibling in the study (youngest excluded), 45 current glucocorticoid medication use, and 4 did not provide any blood or saliva samples. An additional 14 out of 964 plasma samples, and 81 of 871 saliva samples were excluded due to unavailable collection times or samples collected in the afternoon or samples collected more than three hours apart on any of the three days (for saliva only). A total of 1077 eligible individuals remained in the study; n = 948 for plasma and n = 790 for saliva.

### Ethics Statement

Ethics approval was obtained from the Human Ethics Committee at King Edward Memorial Hospital and Princess Margaret Hospital in Perth, Western Australia. Adolescent participants and their guardians provided written consent.

### HPA axis assessment at the 17-year follow-up

As previously described [30], basal HPA activity was measured at the 17-year follow-up using morning fasted 3-day total saliva cortisol, and plasma measures of total cortisol, ACTH, CBG and calculated free cortisol. All samples were collected on weekdays in the home environment following an overnight fast. Study participants were asked to provide saliva samples using Salivette self-collection tubes (Sarstedt, Germany) on three consecutive mornings fifteen minutes after waking and to record the time and date. On the third morning study participants received a home visit from a research nurse and blood was collected at the time of saliva collection. After collection by the research nurse, saliva and plasma were transported, stored and processed at −80°C.

Biochemical analyses of plasma ACTH and total cortisol were conducted at the University of Western Australia and CBG analysis was conducted at Edinburgh University. Cortisol and CBG were measured by ^125^I radioimmunoassay (GamaCoat cortisol RIA-DiaSorin, MN, USA and CBG-RIA-100 – BioSource Europe, SA, Belgium). ACTH was measured by ^125^I immunoradiometric assay (ACTH-IRMA; DiaSorin, MN, USA). Samples were assayed in duplicate and outcomes were re-analyzed with an adjusted sample volume if they were outside the assay standard range. The intra- and inter-assay variations were 8.8% and 13.7% for plasma cortisol (23 assays), 3.9% and 5.5% for CBG (20 assays), and 5.3% and 12.1% for ACTH (19 assays), respectively. Plasma free cortisol levels were calculated using Coolen's equation which takes into account the measured plasma concentrations of total cortisol and CBG, as well as estimated albumin levels [31].

### Exposure Measurement

Birthweight was measured immediately after birth by hospital study personnel for the majority of cases, when the delivery was performed outside KEMH weights were obtained from hospital records. Crude birthweight was used in all analysis. The following covariates were included in all multivariate models: sex, time of blood draw, body mass index (BMI) calculated using weight and height measured by trained research staff at the most recent age 17 follow-up visit, and oral contraceptive use derived from a combination of self-report and confidential interview. Based on known differences in HPA axis activity by estrogen status, a combined variable was created for sex and oral contraceptive use (male, female no oral contraceptive use, and female using oral contraceptive).

### SNP selection and genotyping

Using a candidate gene approach 16 genes that are functionally relevant in the HPA pathway were identified. Common tagging SNPs within coding and regulatory regions with a minor allele frequency (MAF) >10% were selected within these genes. A total of 124 SNPs in the 16 genes were genotyped. Table 1 provides a list of these genes; a list of candidate SNPs is provided in Supplementary Table S1 in File S1.

**Table 1:** List of genes, corresponding gene symbols, and number of SNPs in each gene genotyped in the Western Australia Pregnancy Cohort (Raine) Study.

Gene Name	Gene Symbol	Number of genotyped SNPs	Chromosome
Arginine vasopressin	*AVP*	1	20
Corticotropin releasing hormone	*CRH*	1	8
Corticotropin releasing hormone receptor 1	*CRHR1*	6	17
Corticotropin-releasing hormone receptor 2	*CRHR2*	8	7
Cytochrome P450-11A1	*CYP11A1*	2	15
Cytochrome P450-17A1	*CYP17A1*	2	10
Cytochrome P450-1A2	*CYP21A2*	1	6
Hydroxy-delta-5-steroid dehydrogenase	*HSD3B2*	1	1
Adrenocorticotropic hormone receptor	*MC2R*	5	18
Glucocorticoid receptor	*NR3C1*	8	5
Mineralocorticoid receptor (nuclear receptor subfamily 3)	*NR3C2*	64	4
Oxytocin	*OXT*	3	20
Proopiomelanocortin	*POMC*	3	2
Corticosteroid binding globulin	*SERPINA6*	14	14
Serotonin Transporter	*SLC6A4*	4	17
Urocortin	*UCN*	1	2

DNA was extracted from peripheral blood collected at the year 14 follow-up. DNA was isolated from EDTA-anticoagulated blood samples using Puregene DNA isolation kit. Genotyping was conducted at the Centre for Applied Genomics at the Hospital for Sick Children (Toronto, Ontario, Canada) using the GoldenGate Genotyping Assay (Illumina Inc. San Diego, California, USA). A custom panel was created for the selected SNPs using Illumina's Assay Design Tool (ADT) for probe design (http://www.illumina.com/) and amplified products were scanned using the Illumina BeadArray Reader (Illumina, San Diego, CA), as described previously [32]. Genotypes were analyzed using the Beadstudio v3.0 software and only SNPs with GenCall scores >0.25 were called and samples were discarded if call rates were below 85%. Hardy-Weinberg Equilibrium (HWE) was calculated for each SNP using the R genetics package. Supplementary Table S1 in File S1 presents the results for the tests for HWE; none of the 124 SNPs showed significant departure from HWE at p<0.01.

### Statistical analysis

Multivariate linear regression was used to evaluate the associations between each individual SNP, using an additive model, and the log transformation of each of the 5 HPA outcomes adjusted for age, sex, BMI, time of blood draw, and oral contraceptive use. We also evaluated the associations between birthweight as an exposure and each of the 5 HPA outcomes. For this study, the 5 HPA outcomes evaluated were: plasma measures of basal cortisol, CBG, ACTH, calculated free cortisol, and mean saliva cortisol over the three study days. Gene-birthweight interactions were evaluated for each basal HPA outcome using multivariate linear regression models and the statistical significance of the multiplicative interactions between birthweight and each SNP were calculated. To adjust for multiple testing, we used the simpleM method [33], which calculates the number of independent comparisons and corresponding threshold for statistical significance, set at p = 5×10^−4^ (0.05/100); only slightly less conservative than the threshold obtained by applying the Bonferroni correction for all 124 SNPs (p = 4×10^−4^). Analyses were performed using the statistical software R version 2.14.1.

Multiple SNP analysis was also conducted using a penalized linear regression, the HyperLasso. This method has been described previously [34] and can identify efficiently a subset of SNPs associated with the phenotype of interest, using a penalized likelihood function, when the SNPs are correlated. The HyperLasso uses a Bayesian inspired penalty and while it does not report a p-value for each individual SNP, the penalty function was chosen such that it controls an overall False Discovery Rate (FDR) for the SNP discovery at 5%. The FDR was assessed by simulations based on the following strategy. Our simulations were conducted first by randomly generating 1,000 data sets where we assumed that the response was normally distributed and the SNP panel was identical to our real data with 20 of them associated with the outcome. We also assumed a MAF of 25% and an effect size of 1.2 (with standard deviation of 0.1) under an additive model for all these 20 SNPs. The FDR was then evaluated by estimating the number of false discovered SNPs among all the SNPs found associated with the outcome. The code for the Hyperlasso can be downloaded from: http://www.ebi.ac.uk/projects/BARGEN/.

To further investigate significant SNPs observed in the primary analysis, a secondary analysis was carried out by imputing additional SNPs in significant genes. Imputation was done using Minimac [35] (a low memory, computationally efficient implementation of the MaCH algorithm for genotype imputation) and the 1,000 genomes data as the reference panel. The imputation quality was evaluated, and SNPs with R^2^<0.5 were removed. Haplotype analysis was also conducted based on the imputed dataset. Haplotype block estimation and association analyses were carried out by using Plink.

## Results

A description of the study population and summary of the HPA measures is provided in Table 2. All subjects were close to 17 years of age (range from 16 to 18 years), 549 (51%) were male and 528 (49%) were female. Among females, 342 (65%) reported using oral contraceptives at 17 years of age. The median birth weight was 3400 grams and 80 (7%) children were born preterm. Median ACTH, total cortisol and CBG concentrations were 43.58 (pg/ml), 585.10 (nmol/L) and 46.82 (µg/ml), respectively. The median calculated free plasma cortisol concentration was 35.12 (nmol/L), whereas median saliva free cortisol was 24.60 (nmol/L). Birthweight was not associated with any of the five HPA outcome measures in unadjusted analysis or after adjustment for time of blood draw, sex, oral contraceptive use, maternal pre-pregnancy BMI, maternal smoking during pregnancy and season of blood draw (data not shown).

**Table 2:** Description of Raine Study population and summary of HPA measures.

	No. (%)	Total N
Preterm birth		
Full term	997 (93%)	1077
Preterm (≤37 wks)	80 (7%)	
Sex		
Male	549 (51%)	1077
Female	528 (49%)	
Oral contraceptive use (females only)		
Yes	342 (65%)	528^a^
No	186 (35%)	

	Median (IQR)	
Age (y)	17 (16, 17)	1077
Birth weight (grams)	3400 (3070, 3725)	1076
BMI at age 17 (kg/m^2^)	22.3 (20.1, 24.7)	945
ACTH (pg/ml)	43.58 (33.27, 58.18)	944
Free Cortisol^b^ (nmol/L)	35.12 (24.84, 48.88)	930
Total Cortisol (nmol/L)	585.10 (480.40, 698.30)	947
CBG (µg/ml)	46.82 (40.12, 57.13)	946
Saliva cortisol^c^ (nmol/L)	24.60 (18.76, 32.56)	790

a Oral contraceptive data on females only.

b Calculated free cortisol using Coolen's equation.

c Mean saliva cortisol averaged over three days.

Table 3 shows the adjusted multivariate regression results for all SNPs associated with any of the five HPA outcomes at the nominal p≤0.05. Several SNPs in the *NR3C1*, *NR3C2*, *CRHR1* and *SERPINA6* genes were associated with multiple HPA outcomes at p<0.05; however, none of the observed associations met our criteria for declaring an association based on the adjusted significance level of p<0.0005.

**Table 3:** SNPs associated with log transformed saliva and plasma HPA outcomes with p≤0.05 from multivariate linear regression analysis adjusted for BMI, blood draw time, sex and oral contraception use.

HPA outcome	Gene	SNP	Beta	*P*-value
Calculated Plasma Free Cortisol^a^	*CRHR1*	rs17763104	−0.105	0.023
	*NR3C1*	rs17287745	−0.098	0.035
	*NR3C1*	rs10482642	0.083	0.057
	*CRHR1*	rs171440	0.062	0.049
Plasma Total Cortisol	*SERPINA6*	rs11621961	−0.041	0.059
	*NR3C2*	rs11099694	0.040	0.056
	*NR3C2*	rs13148853	0.053	0.021
	*MC2R*	rs8093607	0.044	0.038
	*CRHR1*	rs17763104	−0.070	0.021
	*NR3C2*	rs2883930	0.060	0.009
	*NR3C1*	rs10482642	0.061	0.030
Plasma CBG	*SERPINA6*	rs11622665	−0.056	0.005
	*SERPINA6*	rs7161521	0.044	0.012
	*SERPINA6*	rs941601	0.061	0.004
	*NR3C2*	rs13105361	0.046	0.012
	*SERPINA6*	rs1956178	−0.053	0.004
	*NR3C2*	rs11724292	−0.047	0.016
	*NR3C2*	rs4635799	0.036	0.018
	*OXT*	rs2770378	−0.033	0.023
Plasma ACTH	*NR3C2*	rs907621	−0.094	0.002
	*NR3C2*	rs17024708	−0.057	0.027
	*NR3C2*	rs5522	−0.101	0.005
	*NR3C2*	rs3846326	−0.078	0.003
	*NR3C2*	rs17484063	−0.067	0.015
	*NR3C2*	rs13148853	0.073	0.003
	*CRHR2*	rs2267710	−0.066	0.005
	*NR3C2*	rs17484601	−0.054	0.017
	*NR3C2*	rs13116099	0.055	0.014
	*CRHR2*	rs2190242	−0.066	0.014
	*CRHR2*	rs2267716	−0.056	0.025
Saliva Cortisol^b^	*NR3C2*	rs879206	0.092	0.010
	*NR3C2*	rs1994624	0.068	0.016
	*OXT*	rs2770378	0.053	0.053
	*NR3C2*	rs3910044	0.057	0.038

a Plasma free cortisol was calculated using Coolen's equation.

b All HPA outcomes are log transformed. Salivary cortisol is the mean concentration over three days.

After running the Hyperlasso, which controls a FDR of 5%, we found that only one SNP was associated with HPA activity in penalized regression analysis, i.e. the SNP rs11621961 in the *SERPINA6* gene (data not shown). This SNP was significantly associated with plasma total cortisol and CBG. No SNPs were associated with ACTH, calculated free cortisol or salivary cortisol. The distribution of plasma CBG and total cortisol concentrations by *SERPINA6* rs11621961 genotype is provided in Table 4. The minor allele is associated with lower levels of each hormone in a dose-response manner; however, only a very small proportion of the variation is explained by this SNP for both total cortisol (r^2^ = 0.006) and CBG (r^2^ = 0.011).

**Table 4:** Distribution of plasma total cortisol and CBG concentrations by genotype for SNP rs11621961, in *SERPINA6*, identified in the HyperLasso Penalized Regression analysis.

Outcome variable	rs11621961 genotype	N	Median (IQR)	Beta^a^	*P*-value
Total plasma cortisol (nmol/L)	CC	340	606.91 (498.10, 715.70)	−0.041	0.059
	CT	370	574.24 (467.50, 678.50)		
	TT	92	558.70 (450.80, 696.50)		
Plasma CBG (µg/ml)	CC	336	48.22 (40.87, 60.41)	−0.028	0.079
	CT	369	45.98 (40.05, 53.62)		
	TT	91	44.88 (39.47, 55.41)		

a Beta and P-value from multivariate linear regression adjusted for for BMI, blood draw time, sex and oral contraception use.

A total of 84 common SNPs in the SERPINA6 gene were imputed. The associations between each imputed SNP and all five HPA outcomes were evaluated and several imputed SNPs in the SERPINA6 gene were significantly associated with plasma CBG levels. After adjustment for multiple comparisons, 8 of the 84 imputed SNPS were significantly associated with CBG (rs2281517, rs941599, rs7161521, rs11622665, rs2144835, rs45515394, rs11621961, rs7146221); the significant SNPs were located in regions across the gene including the promoter and coding regions (Supplementary Table S2 in File S1). Haplotype analyses were conducted to further evaluate the association between genetic variants in SERPINA6 and CBG levels (Supplementary Table S3 in File S1). Six haplotype blocks were identified and at least one haplotype was associated with CBG levels with Bonferroni adjusted p-values approaching statistical significance (p-values between 0.06 and 0.15).

Table 5 presents the results stratified by genotype for the most significant birthweight-SNP interactions with p<0.01. Although the p-values for interaction were <0.01 for several SNPs in each HPA outcome, none of the interactions reach significance after adjustment for multiple comparisons. The p-values for interactions between birthweight and *SERPINA6* rs11621961 (the SNP identified in the penalized regression analysis) were not less than 0.05 for any of the 5 HPA outcome measures.

**Table 5:** The association between birthweight and log transformed saliva and plasma HPA outcomes stratified by genotype for the most significant SNP*birthweight interactions (p<0.01).

HPA outcome	Gene	SNP	Genotype	Beta^a^ for Birthweight (kg) effect size	*P*-value	Interaction *P*-value
Calculated Plasma Free Cortisol^b^	*CRHR1*	rs4458044	GG	0.122	0.049	0.008
			GC	−0.022	0.740	
			CC	−0.292	0.271	
	*SERPINA6*	rs11160169	CC	0.239	0.023	0.008
			CA	−0.004	0.948	
			AA	−0.104	0.330	
Plasma CBG	*NR3C1*	rs2963156	CC	−0.053	0.046	0.006
			CT/TT	0.037	0.313	
Plasma ACTH	*AVP*	rs3761249	TT	0.034	0.329	0.005
			TG/GG	−0.179	0.007	
	*CRHR1*	rs242942	GG	−0.042	0.218	0.006
			GA/AA	0.197	0.006	
Saliva Cortisol^c^	*CRHR2*	rs1076292	CC	−0.043	0.426	0.002
			CG	0.134	0.007	
			GG	0.209	0.123	
	*NR3C2*	rs17484601	TT	−0.056	0.318	0.003
			TG	0.102	0.063	
			GG	0.279	0.013	

a Beta and P-value from multivariate linear regression adjusted for for BMI, blood draw time, sex and oral contraception use.

b Plasma free cortisol was calculated using Coolen's equation.

c All HPA outcomes are log transformed. Salivary cortisol is the mean concentration over three days.

## Discussion

Our study is one of the most comprehensive examinations of the associations between HPA-related gene SNPs and basal HPA activity. To the best of our knowledge, there have been no other studies in adolescents that have investigated the role of genetic variants in HPA function. We evaluated the associations between 124 tag SNPs in candidate genes selected based on their roles in the HPA pathway and plasma measures of total cortisol, CBG, ACTH, calculated free cortisol and salivary cortisol and none of these met the stringent multiple testing criteria for standard linear regression methods. When we analyzed the data using the HyperLasso penalized linear regression, one significant SNP, rs11621961 upstream (1.1 kbp) of the *SERPINA6* gene, was found to be associated with plasma measures of CBG, and total cortisol. Penalized regression methods have been shown to be preferable to single SNP analysis and it has been demonstrated that the HyperLasso method gives the best overall fit compared to other penalization methods [36].

*SERPINA6*, the gene encoding corticosteroid binding globulin (CBG) protein, is the transport protein for glucocorticoids and progestins, and is a member of the serine protease inhibitor (SERPIN) family. Several rare heritable missense mutations have been identified in the CBG gene and are associated with reduced levels of CBG and cortisol [37]. Additional functional SNPs, including rs113418909 and rs28929488, have recently been identified in gene databases (e.g., dbSNP), although limited literature is available on these newly-identified SNPs. Other *SERPINA6* variants have been identified in Chinese populations [38], where *CBG* A51V (rs146744332) heterozygotes were found to have 50% lower CBG levels than non-carriers [38]. It is unknown if the nonsynonymous SNPs previously identified are in linkage disequilibrium (LD) with our significant SNP as the previously identified SNPs are not available in HapMap or 1000 Genomes datasets.

Genetic variation in *SERPINA6* has previously been found to alter glucocorticoid levels in animal studies [39]. In humans, genetic variants in *SERPINA6* have been associated with altered CBG levels or reductions in cortisol binding affinity [37]. Furthermore, rare mutations in *SERPINA6* may be associated with hypertension, fatigue and metabolic syndrome [37]. In addition to rs11621961, when SNPs across the gene were imputed 8 other SNPs in *SERPINA6* were statistically significant after adjustment for multiple comparisons. However, significant associations were only observed for CBG, and not for cortisol or other HPA phenotypes. Haplotype analysis of the *SERPINA6* gene revealed 6 haplotype blocks and within each block at least one haplotype approached significance suggesting that most regions of the gene have potential mutations associated with CBG concentrations.

To the best of our knowledge, our study is the first study to systematically evaluate associations in HPA related genes and basal HPA function in adolescence. Furthermore, our study comprehensively measured HPA function, with basal waking cortisol, ACTH and CBG from blood drawn in the study participants' home. Previous studies of birthweight and cortisol have used blood taken in a clinic setting which is a form of stress test [9]. Despite our detailed measurement of basal HPA activity, no association was found between birthweight and HPA function among this cohort. As reviewed by Reynolds [40], two previous studies found no association between birthweight and unstressed cortisol measures [20], [41] suggesting that birthweight may be associated with stimulated, not basal, HPA function. This is consistent with our results which failed to find an association between birthweight and unstressed HPA activity. Birthweight is not the only marker of adverse exposure in early life and other possible outcomes such as infant growth may be important. Further research is needed to elucidate this association.

We hypothesized that the association between birthweight and HPA activity may be modified by genetic variants (i.e., any association between birthweight and HPA activity may vary by genotype). Although the p-values for some SNP-birthweight interactions were <0.01 for each HPA measure, none remained significant after correction for multiple testing (adjusted significance level of p<4×10^−4^). After stratification by each of these genotypes with interactions p-values<0.01, there was some evidence of effect modification with the direction of the birthweight association differing by genotype; however, these associations were not highly significant and will need to be evaluated in future studies with larger sample sizes.

One previous study has evaluated whether genetic variants modify the association between birthweight and HPA function. Rautenen *et al* found that glucocorticoid haplotypes modified the associations between birth size and plasma and 24-hr salivary cortisol [25]. Despite this, there is previous literature on polymorphisms in HPA genes in relation to HPA activity in adults. The mineralocorticoid receptor (MR) and glucocorticoid receptor (GR) mediate the effect of cortisol on target tissue and stress induced cortisol levels have been associated with polymorphisms in the MR gene (NR3C2) [42]. Elsewhere in a relatively large (n = 1711) and comprehensive candidate gene and GWAS study, SNPs in the FK506 binding protein (FKBP5) gene, which is part of the GR complex, were associated with total daily saliva free cortisol levels [23]. No SNPs in the FKBP5 gene were included in this initial candidate gene study. Further, although no SNPs in POMC gene were associated with HPA measures in our study, a small study in women found an association with the rs1042571 CC genotype in the POMC gene and higher cortisol levels [24]. This POMC SNP was not included in our study, but is in moderately high LD with rs934778 (R^2^ = 0.59), which was not associated with any HPA measures directly in our study, but there was limited evidence of an interaction between this SNP and birthweight (p-value = 0.014).

There are sex-specific differences in HPA activity and it was previously reported in this cohort that plasma CBG, plasma total cortisol and salivary free cortisol concentrations were significantly higher in females than males, and plasma ACTH concentrations were significantly lower in females [30]. We attempted to stratify the results for our primary analysis of genetic variants and HPA outcomes by sex but given the limited sample size the results were difficult to interpret with very large confidence intervals. Future studies with larger sample size are needed to determine if there are sex-specific differences in the associations between HPA pathway genes and basal HPA outcomes. Further, it is unknown if our conclusions among adolescents are generalizable to other age groups.

One of the strengths of our study is the comprehensive measures of basal HPA activity, including plasma cortisol, CBG and ACTH, calculated free cortisol, and measures of saliva cortisol from three days. Plasma was collected in home within 30 minutes of awakening by a research nurse. Most other studies of HPA function measure cortisol only and a clinic visit is required for the blood draw. Despite these strengths there are several challenges in the measurement of HPA activity, in particularly the diurnal variation in cortisol, CBG and ACTH. Given these challenges many studies rely on home collection of saliva cortisol only and it is known that issues with compliance and accuracy of self-reported time of saliva collection affect the validity of saliva cortisol measures [43].

A limitation of genetic studies, such as ours, which evaluate many SNPs, is the potential for false positive results as a result of multiple testing (type I error); we took this into account by using the SimpleM method. Furthermore, using the HyperLasso penalized regression method imposes a prior on the variable selection criteria and is ideal for a large number of SNPs. For the one SNP identified from the penalized regression analysis, we cannot be sure this is the causal SNP; this SNP may be in LD with the true causal SNP. Furthermore, it is a limitation of our study that these results have not been replicated in independent samples. To reduce the potential for population stratification, non-Caucasian study participants were removed from this analysis; this Caucasian population in Western Australia is relatively homogenous. Siblings were also excluded to reduce the potential for systematic bias.

The results of our study suggest that at least one variant in an HPA pathway gene is associated with HPA axis activity among adolescents; however, these results should be considered with caution as independent replication is required. Although no statistically significant interactions held up after adjustment for multiple testing, there is the possibility that associations between adverse exposures in early life, as captured by birthweight and HPA activity may be modified by genotype. These results may contribute to our identification of genetic signatures in early life that predispose risk for disease, and as such have implications for the development of targeted lifestyle or medical intervention efforts. Optimizing these early-life health trajectories is now viewed as a major focus in efforts to reduce the burden of chronic disease (e.g., obesity, cardiovascular disease and mental health conditions), which are occurring at ever increasing rates within our society. Future studies are needed to replicate our results and further inform our understanding of the genetic variants associated with HPA activity.

## Supporting Information

File S1 **File includes Tables S1, S2, and S3.** Supplementary Table S1: Minor Allele Frequency (MAF) and Hardy Weinberg Equilibrium (HWE) for the 124 genotyped tag SNPs in 16 candidate HPA-axis genes. Supplementary Table S2: Associations between imputed SNPs in the SERPINA6 gene and CBG concentrations among adolescents in the Western Australia (Raine) Pregnancy Study. Supplementary Table S3: Haplotype analysis for the association between imputed SNPs in the SERPINA6 gene and CBG concentrations among adolescents in the Western Australia (Raine) Pregnancy Study.
(DOCX)
