# Supplementary material for: Investigation of Genetic Variants, Birthweight and Hypothalamic-Pituitary-Adrenal Axis Function Suggests a Genetic Variant in the SERPINA6 Gene Is Associated with Corticosteroid Binding Globulin in the Western Australia Pregnancy Cohort (Raine) Study
Source: PLoS One. 2014 Apr 1;9(4):e92957. doi: 10.1371/journal.pone.0092957 (PMC3972221; doi:10.1371/journal.pone.0092957)
Supplement: File S1 — File includes Tables S1, S2, and S3. Supplementary Table S1: Minor Allele Frequency (MAF) and Hardy Weinberg Equilibrium (HWE) for the 124 genotyped tag SNPs in 16 candidate HPA-axis genes. Supplementary Table S2: Associations between imputed SNPs in the SERPINA6 gene and CBG concentrations among adolescents in the Western Australia (Raine) Pregnancy Study. Supplementary Table S3: Haplotype analysis for the association between imputed SNPs in the SERPINA6 gene and CBG concentrations among adolescents in the Western Australia (Raine) Pregnancy Study. (DOCX) [file pone.0092957.s001.docx]

**Supporting Information File S1**

**Supplementary Table S1**. Minor Allele Frequency (MAF) and Hardy Weinberg Equilibrium (HWE) for the 124 genotyped tag SNPs in 16 candidate HPA-axis genes.

| **Gene Name** | **Gene Symbol** | **rs Number** | **Chromo-some** | **Major Allele** | **Minor Allele** | **MAF** | **HWE** |
| --- | --- | --- | --- | --- | --- | --- | --- |
| Arginine vasopressin | *AVP* | rs3761249 | 20 | T | G | 0.117 | 0.511 |
| Corticotrophin releasing hormone | *CRH* | rs1870393 | 8 | C | A | 0.172 | 0.721 |
| Corticotropin releasing hormone receptor 1 | *CRHR1* | rs171440 | 17 | C | T | 0.461 | 0.586 |
|  | *CRHR1* | rs17689966 | 17 | G | A | 0.441 | 0.410 |
|  | *CRHR1* | rs17763104 | 17 | G | A | 0.128 | 0.647 |
|  | *CRHR1* | rs242942 | 17 | G | A | 0.104 | 0.718 |
|  | *CRHR1* | rs4458044 | 17 | G | C | 0.247 | 0.715 |
|  | *CRHR1* | rs8072451 | 17 | T | C | 0.237 | 0.452 |
| Corticotropin-releasing hormone receptor 2 | *CRHR2* | rs1076292 | 7 | C | G | 0.358 | 0.105 |
|  | *CRHR2* | rs12701020 | 7 | C | T | 0.185 | 0.113 |
|  | *CRHR2* | rs2190242 | 7 | A | C | 0.215 | 0.689 |
|  | *CRHR2* | rs2267710 | 7 | C | T | 0.315 | 0.432 |
|  | *CRHR2* | rs2267712 | 7 | C | A | 0.127 | 0.217 |
|  | *CRHR2* | rs2267716 | 7 | C | T | 0.251 | 0.102 |
|  | *CRHR2* | rs2284217 | 7 | G | A | 0.200 | 0.341 |
|  | *CRHR2* | rs929377 | 7 | A | T | 0.307 | 0.811 |
| Cytochrome P450-11A1 | *CYP11A1* | rs12917295 | 15 | C | G | 0.393 | 0.669 |
|  | *CYP11A1* | rs1843090 | 15 | G | A | 0.285 | 0.113 |
| Cytochrome P450-17A1 | *CYP17A1* | rs4919686 | 10 | A | C | 0.282 | 0.933 |
|  | *CYP17A1* | rs743572 | 10 | G | A | 0.377 | 0.515 |
| Cytochrome P450-1A2 | *CYP21A2* | rs17421133 | 6 | A | T | 0.316 | 0.695 |
| Hydroxy-delta-5-steroid dehydrogenase | *HSD3B2* | rs17023577 | 1 | G | T | 0.077 | 0.633 |
| Adrenocorticotropic hormone receptor | *MC2R* | rs12954832 | 18 | T | C | 0.191 | 0.098 |
|  | *MC2R* | rs3888305 | 18 | T | A | 0.435 | 0.890 |
|  | *MC2R* | rs8093607 | 18 | T | A | 0.327 | 0.441 |
|  | *MC2R* | rs877127 | 18 | A | T | 0.313 | 0.753 |
|  | *MC2R* | rs9959473 | 18 | G | A | 0.121 | 0.523 |
| Glucocorticoid receptor | *NR3C1* | rs10482616 | 5 | G | A | 0.121 | 0.336 |
|  | *NR3C1* | rs10482642 | 5 | A | G | 0.161 | 0.451 |
|  | *NR3C1* | rs17287745 | 5 | A | G | 0.415 | 0.363 |
|  | *NR3C1* | rs258747 | 5 | C | T | 0.448 | 0.945 |
|  | *NR3C1* | rs2963155 | 5 | A | G | 0.236 | 0.011 |
|  | *NR3C1* | rs2963156 | 5 | C | T | 0.229 | 0.379 |
|  | *NR3C1* | rs4912905 | 5 | G | C | 0.248 | 1.000 |
|  | *NR3C1* | rs9324918 | 5 | A | G | 0.160 | 0.615 |
| Mineralocorticoid receptor (nuclear receptor subfamily 3) | *NR3C2* | rs10004472 | 4 | C | T | 0.173 | 0.154 |
|  | *NR3C2* | rs10010766 | 4 | C | T | 0.359 | 0.605 |
|  | *NR3C2* | rs10026568 | 4 | T | C | 0.445 | 0.891 |
|  | *NR3C2* | rs10031194 | 4 | C | T | 0.208 | 0.918 |
|  | *NR3C2* | rs10032020 | 4 | C | G | 0.179 | **0.049** |
|  | *NR3C2* | rs10050229 | 4 | A | G | 0.261 | 0.927 |
|  | *NR3C2* | rs10519958 | 4 | G | T | 0.140 | 1.000 |
|  | *NR3C2* | rs11099678 | 4 | A | T | 0.371 | 0.771 |
|  | *NR3C2* | rs11099694 | 4 | A | G | 0.339 | 0.198 |
|  | *NR3C2* | rs11724292 | 4 | A | C | 0.185 | 0.737 |
|  | *NR3C2* | rs11737660 | 4 | G | A | 0.230 | 0.503 |
|  | *NR3C2* | rs12331321 | 4 | T | C | 0.119 | 0.747 |
|  | *NR3C2* | rs12511893 | 4 | T | A | 0.150 | 1.000 |
|  | *NR3C2* | rs12647605 | 4 | C | T | 0.437 | 0.534 |
|  | *NR3C2* | rs13105361 | 4 | C | T | 0.197 | 0.085 |
|  | *NR3C2* | rs13116099 | 4 | T | C | 0.372 | 0.884 |
|  | *NR3C2* | rs13116332 | 4 | G | A | 0.352 | 0.552 |
|  | *NR3C2* | rs13133379 | 4 | T | G | 0.206 | 0.351 |
|  | *NR3C2* | rs13137823 | 4 | A | G | 0.212 | 0.263 |
|  | *NR3C2* | rs13148853 | 4 | C | T | 0.247 | 0.236 |
|  | *NR3C2* | rs13150372 | 4 | G | A | 0.293 | 0.806 |
|  | *NR3C2* | rs1355613 | 4 | T | C | 0.253 | 0.179 |
|  | *NR3C2* | rs1429915 | 4 | T | A | 0.426 | 0.627 |
|  | *NR3C2* | rs17024387 | 4 | A | G | 0.294 | 0.142 |
|  | *NR3C2* | rs17024708 | 4 | A | G | 0.232 | 0.848 |
|  | *NR3C2* | rs17483391 | 4 | C | T | 0.140 | 1.000 |
|  | *NR3C2* | rs17483687 | 4 | A | C | 0.071 | 0.795 |
|  | *NR3C2* | rs17483979 | 4 | A | C | 0.217 | 0.618 |
|  | *NR3C2* | rs17484063 | 4 | C | T | 0.188 | 0.318 |
|  | *NR3C2* | rs17484357 | 4 | A | G | 0.166 | 0.805 |
|  | *NR3C2* | rs17484601 | 4 | T | G | 0.372 | 0.562 |
|  | *NR3C2* | rs17620160 | 4 | T | C | 0.125 | 0.757 |
|  | *NR3C2* | rs1879829 | 4 | T | C | 0.443 | 0.945 |
|  | *NR3C2* | rs1994624 | 4 | T | C | 0.380 | 0.472 |
|  | *NR3C2* | rs2883930 | 4 | G | C | 0.256 | 0.594 |
|  | *NR3C2* | rs3846302 | 4 | G | A | 0.141 | 0.578 |
|  | *NR3C2* | rs3846307 | 4 | C | G | 0.341 | 0.406 |
|  | *NR3C2* | rs3846317 | 4 | T | C | 0.378 | 0.470 |
|  | *NR3C2* | rs3846320 | 4 | G | A | 0.197 | 0.830 |
|  | *NR3C2* | rs3846323 | 4 | G | A | 0.319 | 0.184 |
|  | *NR3C2* | rs3846326 | 4 | G | A | 0.213 | 0.761 |
|  | *NR3C2* | rs3910044 | 4 | C | T | 0.410 | 0.183 |
|  | *NR3C2* | rs3910052 | 4 | C | T | 0.341 | 0.705 |
|  | *NR3C2* | rs4635799 | 4 | T | C | 0.494 | 0.342 |
|  | *NR3C2* | rs4835488 | 4 | T | C | 0.457 | 0.837 |
|  | *NR3C2* | rs4835490 | 4 | G | A | 0.194 | 0.447 |
|  | *NR3C2* | rs4835491 | 4 | G | C | 0.297 | 0.122 |
|  | *NR3C2* | rs4835519 | 4 | T | C | 0.350 | 0.179 |
|  | *NR3C2* | rs5522 | 4 | A | G | 0.109 | 0.598 |
|  | *NR3C2* | rs6535579 | 4 | C | T | 0.283 | 0.181 |
|  | *NR3C2* | rs6535594 | 4 | A | G | 0.494 | 0.153 |
|  | *NR3C2* | rs6535598 | 4 | T | G | 0.464 | 0.584 |
|  | *NR3C2* | rs6831034 | 4 | A | T | 0.187 | 0.373 |
|  | *NR3C2* | rs6855341 | 4 | C | T | 0.188 | 0.656 |
|  | *NR3C2* | rs6856424 | 4 | G | C | 0.248 | 0.238 |
|  | *NR3C2* | rs6856803 | 4 | T | C | 0.407 | 0.778 |
|  | *NR3C2* | rs6857487 | 4 | T | A | 0.434 | 0.214 |
|  | *NR3C2* | rs7686433 | 4 | G | A | 0.136 | 0.240 |
|  | *NR3C2* | rs7691663 | 4 | C | A | 0.421 | 0.296 |
|  | *NR3C2* | rs7695118 | 4 | G | A | 0.410 | 0.091 |
|  | *NR3C2* | rs7699349 | 4 | T | C | 0.257 | 0.092 |
|  | *NR3C2* | rs879206 | 4 | A | C | 0.155 | 0.194 |
|  | *NR3C2* | rs907621 | 4 | G | A | 0.161 | 0.315 |
|  | *NR3C2* | rs982076 | 4 | C | T | 0.257 | 0.131 |
| Oxytocin | *OXT* | rs2740210 | 20 | G | T | 0.335 | 1.000 |
|  | *OXT* | rs2770378 | 20 | G | A | 0.428 | 0.067 |
|  | *OXT* | rs6133010 | 20 | G | A | 0.114 | 0.399 |
| Proopiomelanocortin | *POMC* | rs7565427 | 2 | G | A | 0.114 | 0.738 |
|  | *POMC* | rs7565877 | 2 | A | G | 0.108 | 0.111 |
|  | *POMC* | rs934778 | 2 | T | C | 0.306 | 1.000 |
| Corticosteroid binding globulin | *SERPINA6* | rs11160169 | 14 | C | A | 0.461 | 0.152 |
|  | *SERPINA6* | rs11621961 | 14 | C | T | 0.341 | 0.706 |
|  | *SERPINA6* | rs11622665 | 14 | A | G | 0.162 | 0.803 |
|  | *SERPINA6* | rs11629171 | 14 | C | T | 0.298 | 0.685 |
|  | *SERPINA6* | rs1956178 | 14 | G | T | 0.190 | 0.321 |
|  | *SERPINA6* | rs1956179 | 14 | A | G | 0.305 | 0.873 |
|  | *SERPINA6* | rs1998056 | 14 | C | G | 0.445 | 0.243 |
|  | *SERPINA6* | rs2273399 | 14 | C | T | 0.116 | 0.869 |
|  | *SERPINA6* | rs2281519 | 14 | C | T | 0.269 | 0.194 |
|  | *SERPINA6* | rs3790036 | 14 | T | C | 0.183 | 1.000 |
|  | *SERPINA6* | rs7147098 | 14 | G | A | 0.152 | 0.115 |
|  | *SERPINA6* | rs7158343 | 14 | C | G | 0.217 | 0.921 |
|  | *SERPINA6* | rs7161521 | 14 | G | A | 0.226 | 0.699 |
|  | *SERPINA6* | rs941601 | 14 | G | A | 0.146 | 0.133 |
| Serotonin Transporter | *SLC6A4* | rs12150214 | 17 | G | C | 0.185 | 0.498 |
|  | *SLC6A4* | rs140700 | 17 | G | A | 0.092 | 0.038 |
|  | *SLC6A4* | rs4251417 | 17 | G | A | 0.099 | 0.337 |
|  | *SLC6A4* | rs2066713 | 17 | C | T | 0.398 | 0.019 |
| Urocortin | *UCN* | rs4665963 | 2 | C | T | 0.230 | 0.178 |

Supplementary Table S2. Associations between imputed SNPs in the SERPINA6 gene and CBG concentrations among adolescents in the Western Australia (Raine) Pregnancy Study.

| **SNP** | **BP location** | **Minor allele** | **P value** | **FDR adjusted P value** |
| --- | --- | --- | --- | --- |
| rs7146221 | 94769081 | A | 0.0014 | **0.0474** |
| rs11621961 | 94769476 | T | 0.0023 | **0.0474** |
| rs7147098 | 94769817 | A | 0.7554 | 0.8538 |
| rs67922411 | 94769907 | G | 0.0320 | 0.1415 |
| rs11621117 | 94769995 | C | 0.5425 | 0.7591 |
| rs78715582 | 94770184 | C | 0.6080 | 0.7622 |
| rs2273399 | 94770990 | A | 0.2757 | 0.5977 |
| rs45515394 | 94771170 | T | 0.0048 | **0.0474** |
| rs941601 | 94771541 | T | 0.0121 | 0.0680 |
| rs8015996 | 94772157 | A | 0.0908 | 0.3035 |
| rs2228543 | 94772429 | A | 0.0122 | 0.0680 |
| rs1042394 | 94772504 | A | 0.4317 | 0.7110 |
| rs72704334 | 94772851 | C | 0.0122 | 0.0680 |
| rs72704335 | 94772886 | T | 0.3372 | 0.6811 |
| rs72704338 | 94772925 | C | 0.0122 | 0.0680 |
| rs3790036 | 94773121 | G | 0.3057 | 0.6419 |
| rs142835944 | 94773129 | C | 0.3711 | 0.7085 |
| rs3790035 | 94773242 | T | 0.0908 | 0.3035 |
| rs12437272 | 94773371 | T | 0.0122 | 0.0680 |
| rs12437224 | 94773395 | T | 0.0121 | 0.0680 |
| rs2144835 | 94773411 | C | 0.0047 | **0.0474** |
| rs11629171 | 94773450 | T | 0.0908 | 0.3035 |
| rs2144834 | 94773634 | A | 0.4299 | 0.7110 |
| rs2144833 | 94773788 | T | 0.1662 | 0.4531 |
| rs8023023 | 94773876 | A | 0.5531 | 0.7591 |
| rs8022616 | 94773945 | G | 0.2447 | 0.5758 |
| rs2180401 | 94774017 | T | 0.5531 | 0.7591 |
| rs59173829 | 94774023 | T | 0.4317 | 0.7110 |
| rs61980587 | 94774186 | C | 0.4317 | 0.7110 |
| rs2144832 | 94774192 | C | 0.5531 | 0.7591 |
| rs1950661 | 94774298 | C | 0.5531 | 0.7591 |
| rs1950660 | 94774300 | A | 0.5531 | 0.7591 |
| rs11622665 | 94774995 | G | 0.0038 | **0.0474** |
| rs10498639 | 94775526 | A | 0.5964 | 0.7591 |
| rs11627651 | 94775687 | C | 0.5964 | 0.7591 |
| rs11623055 | 94775701 | A | 0.5964 | 0.7591 |
| rs71431631 | 94775707 | T | 0.3991 | 0.7110 |
| rs11622970 | 94775737 | G | 0.5964 | 0.7591 |
| rs72704349 | 94775926 | G | 0.9867 | 0.9867 |
| rs2228542 | 94776219 | T | 0.4021 | 0.7110 |
| rs2228541 | 94776221 | C | 0.5964 | 0.7591 |
| rs2281520 | 94776387 | G | 0.4021 | 0.7110 |
| rs11160168 | 94776401 | T | 0.1727 | 0.4533 |
| rs11160169 | 94776441 | A | 0.5964 | 0.7591 |
| rs2281519 | 94776632 | A | 0.5415 | 0.7591 |
| rs149108558 | 94776771 | T | 0.9867 | 0.9867 |
| rs72704352 | 94776994 | A | 0.1672 | 0.4531 |
| rs10141488 | 94777280 | A | 0.9867 | 0.9867 |
| rs10141408 | 94777361 | T | 0.9867 | 0.9867 |
| rs8005533 | 94777406 | C | 0.5964 | 0.7591 |
| rs8005719 | 94777500 | T | 0.1350 | 0.3911 |
| rs112209637 | 94778064 | G | 0.9867 | 0.9867 |
| rs35087450 | 94778307 | G | 0.3405 | 0.6811 |
| rs10144771 | 94778653 | A | 0.0699 | 0.2936 |
| rs72704356 | 94778663 | T | 0.9867 | 0.9867 |
| rs72704357 | 94778878 | A | 0.9867 | 0.9867 |
| rs28515150 | 94779252 | G | 0.9867 | 0.9867 |
| rs72704358 | 94779725 | T | 0.7981 | 0.8821 |
| rs3748320 | 94780608 | A | 0.7052 | 0.8459 |
| rs11626522 | 94781461 | A | 0.0879 | 0.3035 |
| rs7154770 | 94782798 | C | 0.7052 | 0.8459 |
| rs7158343 | 94783153 | G | 0.7052 | 0.8459 |
| rs2092977 | 94783172 | A | 0.2536 | 0.5758 |
| rs72704359 | 94783780 | C | 0.7624 | 0.8538 |
| rs12590834 | 94783793 | A | 0.0134 | 0.0705 |
| rs72704360 | 94784123 | T | 0.7624 | 0.8538 |
| rs61980636 | 94784618 | T | 0.0183 | 0.0854 |
| rs11627241 | 94785451 | T | 0.2536 | 0.5758 |
| rs72704361 | 94785459 | T | 0.7624 | 0.8538 |
| rs1956179 | 94785742 | C | 0.1255 | 0.3764 |
| rs1956178 | 94786544 | A | 0.0183 | 0.0854 |
| rs78739778 | 94787072 | G | 0.2536 | 0.5758 |
| rs7161521 | 94787288 | T | 0.0051 | **0.0474** |
| rs34097828 | 94787674 | G | 0.5187 | 0.7591 |
| rs941600 | 94788310 | T | 0.2536 | 0.5758 |
| rs941599 | 94788341 | T | 0.0051 | **0.0474** |
| rs182300444 | 94788509 | G | 0.0794 | 0.3035 |
| rs4900225 | 94788593 | A | 0.7150 | 0.8459 |
| rs4905177 | 94788715 | T | 0.2775 | 0.5977 |
| rs2281518 | 94789117 | G | 0.0051 | **0.0474** |
| rs1998057 | 94789192 | C | 0.1240 | 0.3764 |
| rs56252611 | 94789223 | A | 0.0939 | 0.3035 |
| rs1998056 | 94789495 | C | 0.3613 | 0.7058 |
| rs2281517 | 94789787 | G | 0.0051 | **0.0474** |

Supplementary Table S3. Haplotype analysis for the association between imputed SNPs in the SERPINA6 gene and CBG concentrations among adolescents in the Western Australia (Raine) Pregnancy Study.

| **LD block** | **Haplotype** | **Freq** | **SNPs in Haplotype** | **P value** | **Bonferroni adjusted p-value** |
| --- | --- | --- | --- | --- | --- |
| H1 | ACG | 0.0126 | rs7146221\|rs11621961\|rs7147098 | 0.2545 | 1.0000 |
| H1 | ATG | 0.3436 | rs7146221\|rs11621961\|rs7147098 | 0.0031 | **0.1010** |
| H1 | GCA | 0.1501 | rs7146221\|rs11621961\|rs7147098 | 0.7554 | 1.0000 |
| H1 | GCG | 0.4912 | rs7146221\|rs11621961\|rs7147098 | 0.0035 | 0.1151 |
| H2 | ACT | 0.1489 | rs67922411\|rs11621117\|rs78715582 | 0.0019 | **0.0616** |
| H2 | ATC | 0.1495 | rs67922411\|rs11621117\|rs78715582 | 0.6080 | 1.0000 |
| H2 | ATT | 0.5320 | rs67922411\|rs11621117\|rs78715582 | 0.3435 | 1.0000 |
| H2 | GCT | 0.1696 | rs67922411\|rs11621117\|rs78715582 | 0.0320 | 1.0000 |
| H3 | GCAGGTTATGCTTGGAGTATCCAAACACGCCACAG | 0.1156 | rs45515394\|rs941601\|rs8015996\|rs2228543\|rs1042394\|rs72704334\|rs72704338\|rs3790036\|rs3790035\|rs12437272\|rs12437224\|rs2144835\|rs11629171\|rs2144834\|rs2144833\|rs8023023\|rs8022616\|rs2180401\|rs59173829\|rs61980587\|rs2144832\|rs1950661\|rs1950660\|rs11622665\|rs10498639\|rs11627651\|rs11623055\|rs71431631\|rs11622970\|rs2228542\|rs2228541\|rs2281520\|rs11160168\|rs11160169\|rs2281519 | 0.2051 | 1.0000 |
| H3 | GCAGGTTGTGCTTGGAATATCCAAACACGCCACAA | 0.1846 | rs45515394\|rs941601\|rs8015996\|rs2228543\|rs1042394\|rs72704334\|rs72704338\|rs3790036\|rs3790035\|rs12437272\|rs12437224\|rs2144835\|rs11629171\|rs2144834\|rs2144833\|rs8023023\|rs8022616\|rs2180401\|rs59173829\|rs61980587\|rs2144832\|rs1950661\|rs1950660\|rs11622665\|rs10498639\|rs11627651\|rs11623055\|rs71431631\|rs11622970\|rs2228542\|rs2228541\|rs2281520\|rs11160168\|rs11160169\|rs2281519 | 0.2675 | 1.0000 |
| H3 | GCGGATTACGCTCAGGAGTCTAGAGTGTATAGCCG | 0.2236 | rs45515394\|rs941601\|rs8015996\|rs2228543\|rs1042394\|rs72704334\|rs72704338\|rs3790036\|rs3790035\|rs12437272\|rs12437224\|rs2144835\|rs11629171\|rs2144834\|rs2144833\|rs8023023\|rs8022616\|rs2180401\|rs59173829\|rs61980587\|rs2144832\|rs1950661\|rs1950660\|rs11622665\|rs10498639\|rs11627651\|rs11623055\|rs71431631\|rs11622970\|rs2228542\|rs2228541\|rs2281520\|rs11160168\|rs11160169\|rs2281519 | 0.4413 | 1.0000 |
| H3 | GCGGATTACGCTCGGGAGTCTAGAGTGCATAGCCG | 0.0251 | rs45515394\|rs941601\|rs8015996\|rs2228543\|rs1042394\|rs72704334\|rs72704338\|rs3790036\|rs3790035\|rs12437272\|rs12437224\|rs2144835\|rs11629171\|rs2144834\|rs2144833\|rs8023023\|rs8022616\|rs2180401\|rs59173829\|rs61980587\|rs2144832\|rs1950661\|rs1950660\|rs11622665\|rs10498639\|rs11627651\|rs11623055\|rs71431631\|rs11622970\|rs2228542\|rs2228541\|rs2281520\|rs11160168\|rs11160169\|rs2281519 | 0.9375 | 1.0000 |
| H3 | GCGGGTTACGCCCGGAATATCCAGACACGCCACAA | 0.0868 | rs45515394\|rs941601\|rs8015996\|rs2228543\|rs1042394\|rs72704334\|rs72704338\|rs3790036\|rs3790035\|rs12437272\|rs12437224\|rs2144835\|rs11629171\|rs2144834\|rs2144833\|rs8023023\|rs8022616\|rs2180401\|rs59173829\|rs61980587\|rs2144832\|rs1950661\|rs1950660\|rs11622665\|rs10498639\|rs11627651\|rs11623055\|rs71431631\|rs11622970\|rs2228542\|rs2228541\|rs2281520\|rs11160168\|rs11160169\|rs2281519 | 0.0137 | 0.4524 |
| H3 | GCGGGTTACGCCCGGAATATCCAGACACGCCACAG | 0.0747 | rs45515394\|rs941601\|rs8015996\|rs2228543\|rs1042394\|rs72704334\|rs72704338\|rs3790036\|rs3790035\|rs12437272\|rs12437224\|rs2144835\|rs11629171\|rs2144834\|rs2144833\|rs8023023\|rs8022616\|rs2180401\|rs59173829\|rs61980587\|rs2144832\|rs1950661\|rs1950660\|rs11622665\|rs10498639\|rs11627651\|rs11623055\|rs71431631\|rs11622970\|rs2228542\|rs2228541\|rs2281520\|rs11160168\|rs11160169\|rs2281519 | 0.1672 | 1.0000 |
| H3 | GCGGGTTACGCCCGTGAGATTAGAGTGCACAATCG | 0.1489 | rs45515394\|rs941601\|rs8015996\|rs2228543\|rs1042394\|rs72704334\|rs72704338\|rs3790036\|rs3790035\|rs12437272\|rs12437224\|rs2144835\|rs11629171\|rs2144834\|rs2144833\|rs8023023\|rs8022616\|rs2180401\|rs59173829\|rs61980587\|rs2144832\|rs1950661\|rs1950660\|rs11622665\|rs10498639\|rs11627651\|rs11623055\|rs71431631\|rs11622970\|rs2228542\|rs2228541\|rs2281520\|rs11160168\|rs11160169\|rs2281519 | 0.5172 | 1.0000 |
| H3 | GTGAGCCACTTTCGTGAGATTAGAGTGCACAATCG | 0.0151 | rs45515394\|rs941601\|rs8015996\|rs2228543\|rs1042394\|rs72704334\|rs72704338\|rs3790036\|rs3790035\|rs12437272\|rs12437224\|rs2144835\|rs11629171\|rs2144834\|rs2144833\|rs8023023\|rs8022616\|rs2180401\|rs59173829\|rs61980587\|rs2144832\|rs1950661\|rs1950660\|rs11622665\|rs10498639\|rs11627651\|rs11623055\|rs71431631\|rs11622970\|rs2228542\|rs2228541\|rs2281520\|rs11160168\|rs11160169\|rs2281519 | 0.6959 | 1.0000 |
| H3 | TTGAGCCACTTTCGTGAGATTAGAGTGCACAATCG | 0.1231 | rs45515394\|rs941601\|rs8015996\|rs2228543\|rs1042394\|rs72704334\|rs72704338\|rs3790036\|rs3790035\|rs12437272\|rs12437224\|rs2144835\|rs11629171\|rs2144834\|rs2144833\|rs8023023\|rs8022616\|rs2180401\|rs59173829\|rs61980587\|rs2144832\|rs1950661\|rs1950660\|rs11622665\|rs10498639\|rs11627651\|rs11623055\|rs71431631\|rs11622970\|rs2228542\|rs2228541\|rs2281520\|rs11160168\|rs11160169\|rs2281519 | 0.0048 | 0.1573 |
| H4 | ACGTGGATCGGCCCCCCCC | 0.0685 | rs72704352\|rs8005533\|rs8005719\|rs35087450\|rs10144771\|rs3748320\|rs11626522\|rs7154770\|rs7158343\|rs2092977\|rs12590834\|rs61980636\|rs11627241\|rs1956179\|rs1956178\|rs78739778\|rs7161521\|rs34097828\|rs941600 | 0.0639 | 1.0000 |
| H4 | GCGTGGGTCGACCCCCTCC | 0.1267 | rs72704352\|rs8005533\|rs8005719\|rs35087450\|rs10144771\|rs3748320\|rs11626522\|rs7154770\|rs7158343\|rs2092977\|rs12590834\|rs61980636\|rs11627241\|rs1956179\|rs1956178\|rs78739778\|rs7161521\|rs34097828\|rs941600 | 0.0196 | 0.6461 |
| H4 | GCGTGGGTCGGCCCCCTCC | 0.0905 | rs72704352\|rs8005533\|rs8005719\|rs35087450\|rs10144771\|rs3748320\|rs11626522\|rs7154770\|rs7158343\|rs2092977\|rs12590834\|rs61980636\|rs11627241\|rs1956179\|rs1956178\|rs78739778\|rs7161521\|rs34097828\|rs941600 | 0.2618 | 1.0000 |
| H4 | GCGTGGGTCGGTCTACCCC | 0.1616 | rs72704352\|rs8005533\|rs8005719\|rs35087450\|rs10144771\|rs3748320\|rs11626522\|rs7154770\|rs7158343\|rs2092977\|rs12590834\|rs61980636\|rs11627241\|rs1956179\|rs1956178\|rs78739778\|rs7161521\|rs34097828\|rs941600 | 0.0103 | 0.3386 |
| H4 | GTGGGAGCGGGCCTCCCGC | 0.2104 | rs72704352\|rs8005533\|rs8005719\|rs35087450\|rs10144771\|rs3748320\|rs11626522\|rs7154770\|rs7158343\|rs2092977\|rs12590834\|rs61980636\|rs11627241\|rs1956179\|rs1956178\|rs78739778\|rs7161521\|rs34097828\|rs941600 | 0.4266 | 1.0000 |
| H4 | GTGGGGGTCGGTCTACCCC | 0.0190 | rs72704352\|rs8005533\|rs8005719\|rs35087450\|rs10144771\|rs3748320\|rs11626522\|rs7154770\|rs7158343\|rs2092977\|rs12590834\|rs61980636\|rs11627241\|rs1956179\|rs1956178\|rs78739778\|rs7161521\|rs34097828\|rs941600 | 0.4116 | 1.0000 |
| H4 | GTGTGGGTCGGCCTCCCCC | 0.0245 | rs72704352\|rs8005533\|rs8005719\|rs35087450\|rs10144771\|rs3748320\|rs11626522\|rs7154770\|rs7158343\|rs2092977\|rs12590834\|rs61980636\|rs11627241\|rs1956179\|rs1956178\|rs78739778\|rs7161521\|rs34097828\|rs941600 | 0.9867 | 1.0000 |
| H4 | GTTTAGGTCAGCTTCGCCT | 0.2362 | rs72704352\|rs8005533\|rs8005719\|rs35087450\|rs10144771\|rs3748320\|rs11626522\|rs7154770\|rs7158343\|rs2092977\|rs12590834\|rs61980636\|rs11627241\|rs1956179\|rs1956178\|rs78739778\|rs7161521\|rs34097828\|rs941600 | 0.1427 | 1.0000 |
| H5 | CA | 0.2682 | rs941599\|rs4900225 | 0.0023 | 0.0754 |
| H5 | CC | 0.5031 | rs941599\|rs4900225 | 0.7150 | 1.0000 |
| H5 | TA | 0.2286 | rs941599\|rs4900225 | 0.0051 | 0.1676 |
| H6 | CAAGCA | 0.1916 | rs4905177\|rs2281518\|rs1998057\|rs56252611\|rs1998056\|rs2281517 | 0.0183 | 0.6039 |
| H6 | CACAGA | 0.0760 | rs4905177\|rs2281518\|rs1998057\|rs56252611\|rs1998056\|rs2281517 | 0.0940 | 1.0000 |
| H6 | CACGGA | 0.2569 | rs4905177\|rs2281518\|rs1998057\|rs56252611\|rs1998056\|rs2281517 | 0.5182 | 1.0000 |
| H6 | CGAGGG | 0.2286 | rs4905177\|rs2281518\|rs1998057\|rs56252611\|rs1998056\|rs2281517 | 0.0051 | 0.1676 |
| H6 | TAAGCA | 0.2469 | rs4905177\|rs2281518\|rs1998057\|rs56252611\|rs1998056\|rs2281517 | 0.2775 | 1.0000 |
